# Genome-Wide Association Study Identifies Loci and Candidate Genes for Body Composition and Meat Quality Traits in Beijing-You Chickens

**DOI:** 10.1371/journal.pone.0061172

**Published:** 2013-04-18

**Authors:** Ranran Liu, Yanfa Sun, Guiping Zhao, Fangjie Wang, Dan Wu, Maiqing Zheng, Jilan Chen, Lei Zhang, Yaodong Hu, Jie Wen

**Affiliations:** 1 Institute of Animal Sciences, Chinese Academy of Agricultural Sciences, Beijing, P.R. China; 2 State Key Laboratory of Animal Nutrition, Beijing, P. R. China; 3 College of Animal Science and Technology, Yangzhou University, Yangzhou, Jiangsu, P. R. China; 4 College of Animal Science and Technology, Anhui Agricultural University, Hefei, Anhui, P. R. China; University of Illinois, United States of America

## Abstract

Body composition and meat quality traits are important economic traits of chickens. The development of high-throughput genotyping platforms and relevant statistical methods have enabled genome-wide association studies in chickens. In order to identify molecular markers and candidate genes associated with body composition and meat quality traits, genome-wide association studies were conducted using the Illumina 60 K SNP Beadchip to genotype 724 Beijing-You chickens. For each bird, a total of 16 traits were measured, including carcass weight (CW), eviscerated weight (EW), dressing percentage, breast muscle weight (BrW) and percentage (BrP), thigh muscle weight and percentage, abdominal fat weight and percentage, dry matter and intramuscular fat contents of breast and thigh muscle, ultimate pH, and shear force of the *pectoralis major* muscle at 100 d of age. The SNPs that were significantly associated with the phenotypic traits were identified using both simple (GLM) and compressed mixed linear (MLM) models. For nine of ten body composition traits studied, SNPs showing genome wide significance (P<2.59E−6) have been identified. A consistent region on chicken (Gallus gallus) chromosome 4 (GGA4), including seven significant SNPs and four candidate genes (*LCORL*, *LAP3*, *LDB2*, *TAPT1*), were found to be associated with CW and EW. Another 0.65 Mb region on GGA3 for BrW and BrP was identified. After measuring the mRNA content in beast muscle for five genes located in this region, the changes in *GJA1* expression were found to be consistent with that of breast muscle weight across development. It is highly possible that *GJA1* is a functional gene for breast muscle development in chickens. For meat quality traits, several SNPs reaching suggestive association were identified and possible candidate genes with their functions were discussed.

## Introduction

With the advance of high-throughput genotyping platforms, much effort has been spent on identifying molecular markers and genes related to complex traits using genome-wide association studies (GWAS) in several species.

In the field of animal breeding, loci or narrow regions affecting milk production, fertility and growth traits in cattle [1]–[3], body composition, intramuscular fat content, meat color in pigs [4], [5] and growth and egg quality in chickens [6], [7] have been detected successfully using genome-wide association studies. Such information helps in the development of marker assisted breeding as well as by improving understanding of the molecular mechanisms underlying the target traits.

Body composition and meat quality in broilers are important economic traits. Body composition traits have been analyzed using QTL techniques in F2 crosses between various lines of chickens. A total of 146 QTLs reaching significance have been reported via genome scans based on marker-QTL linkage analyses (http://www.animalgenome.org/cgi-bin/QTLdb/GG/index,July,2012), which associated with eight body composition traits: carcass weight, dressed percentage, weight and percentage of breast muscle, thigh muscle and abdominal fat. Similar studies on meat quality traits are rare except for 10 QTLs reported for pH value. Application of these QTL results in broiler breeding remains impracticable because of the low precision of mapping.

In the present work, with the aim of identifying potential loci and candidate genes affecting body composition and meat quality traits, 724 chickens from a conservation population of a typical Chinese local breed (Beijing-You chicken) were used in GWAS studies. A total of 16 body composition and meat quality traits were measured or derived.

## Materials and Methods

### Ethics Statement

The animal component of this study was conducted in accordance with the Guidelines for the Experimental Animals, established by the Ministry of Science and Technology (Beijing, China). Blood was collected from the brachial vein of the chickens at 80 d by venipuncture, using citrated syringes during a routine health inspection at the experimental station of the Chinese Academy of Agricultural Sciences (CAAS). Animal experiments were approved by the Science Research Department (in charge of animal welfare issue) of the Institute of Animal Sciences, CAAS (Beijing, China).

### Birds and Phenotypic Traits

The 728 male Beijing-You chickens were generated from 50 half-sib families. The 50 sires and 250 dams were chosen from conservation stock for this breed and were unrelated. The Beijing-You chickens maintained by the Institute of Animal Sciences, CAAS and have not been subjected to any systematic selection for any trait. All birds were reared in stair-step caging under the same recommended nutritional and environmental conditions. A total of 16 carcass and meat quality traits were measured for the GWAS studies: carcass weight (including feet and head, CW), eviscerated weight (EW), breast weight (BrW), thigh muscle weight (ThW), abdominal fat weight (AbFW), dressed percentage (DP), eviscerated yield, as a percentage of CW (EWP), BrW, ThW and AbFW as percentages of EW (BrP, ThP, AbFP), dry matter and intramuscular fat contents of breast and thigh muscle (DM_Br_, DM_Th_, IMF_Br_ and IMF_Th_), ultimate pH (pHu), and shear force (SF) of the *pectoralis major* muscle. The measurements and data were obtained as follows:

After a 12-h fast, birds were weighed (LW) and slaughtered at d 100 by standard commercial procedures and CW was recorded. The removable adipose tissues surrounding the proventriculus and gizzard along with those located around the cloaca were weighed as AbFW [8], [9]. Then EW was weighed. Carcasses were then dissected into deboned, skinless thighs and breasts for weighing and storage at −20°C until used.

The data for CW and EW were also expressed as percentages on the basis of LW and CW, respectively (DP and EWP). The pHu was the mean of three measurements taken from the left breast muscle after 24-h storage at 4°C (IQ150 pH meter (Hach Company, Loveland, USA). Shear force was determined on breast muscles following Li’s method [10] using a universal Warner-Bratzler testing machine MTS Synergie 200 (G-R Manufacturing Company, Manhattan, KS). Dry matter (DM) and IMF in muscle were determined for 20 g samples of the right breast and thigh after removing obvious fat, mincing, and drying in two stages (∼12 h each at 65°C then 105°C, as described by Cui et al [11]. The DM content was expressed on the basis of fresh muscle weight. The IMF was measured by Soxhlet extraction with anhydrous diethyl ether and was expressed as a percentage of muscle DM.

One-day-old hatchlings with similar genetic background were reared in the same conditions as above. On each of weeks 4, 8, 10, 12, and 14, six to eight birds of similar weight were randomly selected, stunned, and euthanized using approved procedures. Breast muscle was rapidly dissected, weighed, snap-frozen in liquid nitrogen, and stored at −80°C for mRNA extraction.

### Genotyping and Quality Control

Genomic DNA was extracted from the blood samples using phenol-chloroform and was diluted to 50 ng/µl. Each chicken was genotyped using the Illumina 60 K Chicken SNP Beadchip (DNA LandMarks Inc., Saint-Jean-sur-Richelieu, Quebec, Canada). Of the total of 728 chickens, four were excluded because sample call rate was <95%. Approximately 20% (12,088) of the SNPs were removed for one or more of the following: low call rate (<95%), minor allelic frequency (<0.01) and Hardy-Weinberg equilibrium (HWE) test (p<1E−06), or chromosomal location was unknown. After imposing these constraints, 724 individuals and 45,548 SNP markers that distributed on 30 autosomes and the Z chromosome (Table 1) were used for the genome-wide association analyses.

**Table 1:** Distributions of SNPs after quality control and the average distances between adjacent SNPs on each chromosome.

Chromosome	Physical Map (Mb)^1^	No. of SNP markers	Average distance (kb)
1	200.9	7224	27.81
2	154.8	5419	28.57
3	113.6	4101	27.7
4	94.2	3294	28.6
5	62.2	2205	28.21
6	37.3	1680	22.2
7	38.3	1809	21.17
8	30.6	1376	22.24
9	25.5	1190	21.43
10	22.5	1307	17.21
11	21.9	1196	18.31
12	20.5	1363	15.04
13	18.9	1129	16.74
14	15.8	1034	15.28
15	13	1017	12.78
16	0.4	21	19.05
17	11.2	864	12.96
18	10.9	831	13.12
19	9.9	825	12
20	13.9	1493	9.31
21	6.9	794	8.69
22	3.9	277	14.08
23	6	619	9.69
24	6.4	684	9.36
25	2	176	11.36
26	5.1	646	7.89
27	4.7	451	10.42
28	4.5	556	8.09
E22^2^	0.9	103	8.74
E64^2^	0.05	3	16.61
Z	74.6	1861	40.09
Total	10131.4	45548	534.75

Note: ^1^physical length of the chromosome was based on the position of the last marker in the WASHUC2 build;

2 E22 and E64 are linkage groups.

### Statistical Analysis

The SNPs that were significantly associated with the phenotypic traits were identified using both simple (GLM, **I**) and compressed mixed linear (MLM, **II**) models [12]:
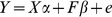
(1)

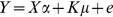
(2)where *Y* is the phenotypic value, *X* is the genotype (45,548 SNPs), *F* is the family, and *K* is the relative kinship matrix; *X*α were regarded as fixed effects, while *Fβ* and *Kμ* were random effects, and *e* is the random error.

The relative kinship matrix (*K*) was constructed from 8,006 independent SNP markers, acquired using Plink v1.07 software [13] through all autosomal SNPs, pruned using the indep-pairwise option, with a window size of 25 SNPs, a step of 5 SNPs, and r^2^ threshold of 0.2. The analyses were performed using TASSEL 3.0 software [14].

The raw data for some traits (AbFP, AbFW, IMF_Br_, DM_Th_, pHu and SF) deviated from normality and Box-Cox or Johnson transformations were applied using Minitab 15 (http://www.minitab.com). Significance thresholds were established from the estimated number of independent SNP markers and LD blocks, defined as a set of contiguous SNPs having pairwise r^2^ values >0.40. Using this approach, the estimated total number of independent SNP markers and LD blocks was 19,284. The two threshold P-values were therefore set at 5.19E−05 (1/19,284) for suggestive significance, and 2.59E−06 (0.05/19,284) for genome-wide significance [15].

Quantile-quantile (Q-Q) plots for each trait and Manhattan plots of genome-wide association analyses were produced with R 2.13.2 software (http://www.r-project.org/).

### Quantitative Measurement of mRNA

The methods for quantitative measurement of mRNA were referred to Li’s methods [10]. Primers for the genes *NCOA7*, *TPD52L1*, *FABP7*, *GJA1* and *ASF1A* were designed (Primer Premier 5.0) from the GenBank sequences (Table 2).

**Table 2:** The specific primers for RT-PCR and q-PCR in this study.

Gene	Sequence	Productsize (bp)	Cycle profile	Accession number
*FABP7*	F: 5′-CGTGATCAGGACTCAGAGCA-3′R: 5′-TCTCTTTGCCATCCCATTTC-3′	158	95°C for 30 s,95°C for 5 s and60°C for 32 s (40 cycles)	NM_205308.2
*TPD52L1*	F: 5′-TCAGCGTACAAGAAGACGCA-3′R: 5′-GGCATGCTTATGGAATGGCG-3′	152	95°C for 30 s,95°C for 5 s and60°C for 32 s (40 cycles)	NM_204215.1
*NCOA7*	F: 5′-CAATTGTTCCAGGCCAGATT-3′R: 5′-TCTTGCCAAATCAGCATCAG-3′	137	95°C for 30 s,95°C for 5 s and60°C for 32 s (40 cycles)	NM_001012878.1
*GJA1*	F: 5′-CATCAGCAGCGCCAATATC-3′R: 5′-TTCATCTCCCCAAGCAGACT-3′	171	95°C for 30 s,95°C for 5 s and60°C for 32 s (40 cycles)	NM_204586.2
*ASF1A*	F: 5′-GACCTGTCGGAAGATTTGGA-3′R: 5′-GGAATAAGCCCTGGGTTAGG-3′	158	95°C for 30 s,95°C for 5 s and60°C for 32 s (40 cycles)	NM_001044690.1
*β-actin*	F: 5′-GAGAAATTGTGCGTGACATCA-3′R: 5′-CCTGAACCTCTCATTGCCA-3′	152	95°C for 30 s,95°C for 5 s and60°C for 32 s (40 cycles)	NM_205518

## Results

Descriptive statistics of the phenotypic measurements of body composition and meat quality traits in the 724 Beijing-You chickens used for the present GWAS studies are given in Table 3. All non-normal phenotypic data except those for pHu were normalized after the Box-Cox or Johnson transformation (Table 4).

**Table 3:** Descriptive statistics of phenotypic data.

Traits	Mean	Standard deviation	Minimum	Maximum	1CV
Live weight (LW, g)	1500.90	176.10	802.00	1996.00	11.73
Carcass weight (CW, g)	1222.50	137.60	786.00	1702.00	11.25
Eviscerated weight (EW, g)	1024.80	121.30	666.00	1458.10	11.84
Dressed percentage (DP, %)	81.38	3.17	71.29	92.44	3.90
Percentage of eviscerated yield (EWP, %)	68.11	2.97	60.21	79.05	4.36
Breast muscle weight (BrW, g)	140.10	24.26	64.00	228.00	17.32
Percentage of breast muscle (BrP, %)	13.63	1.57	7.64	17.50	11.48
Thigh muscle weight (ThW, g)	205.97	34.69	100.00	356.00	16.84
Percentage of thigh muscle (ThP, %)	20.06	2.03	11.84	27.57	10.10
Weight of abdominal fat (AbFW, g)	10.60	8.38	0.17	48.50	79.06
Percentage of abdominal fat (AbFP, %)	1.01	0.77	0.02	4.36	75.87
Dry matter content in breast (DM_Br_, %)	28.16	0.96	25.32	31.66	3.40
Intramuscular fat in breast (IMF_Br_, %)	2.43	0.88	0.57	7.20	36.10
Dry matter content in thigh (DM_Th_, %)	25.21	1.05	22.12	28.29	4.16
Intramuscular fat in thigh (IMF_Th_, %)	9.42	3.09	2.10	20.49	32.77
Ultimate pH (pHu)	5.27	0.19	4.67	6.07	3.65
Shear force of breast muscle (SF, %)	5.02	1.32	1.71	1.01	26.28

1 CV: coefficient of variation.

**Table 4:** Phenotypic mean, standard deviation and status of normalization for non-normal traits after the transformation.

Traits	Mean	Standarddeviation	Status ofnormalization
AbFW	2.62E−02	0.94	Yes
AbFP	3.29E−02	0.97	Yes
IMF_Br_	8.93E−01	0.41	Yes
DM_Th_	5.20E−03	0.99	Yes
SF	2.14E−02	1.02	Yes

**Figure 1:**
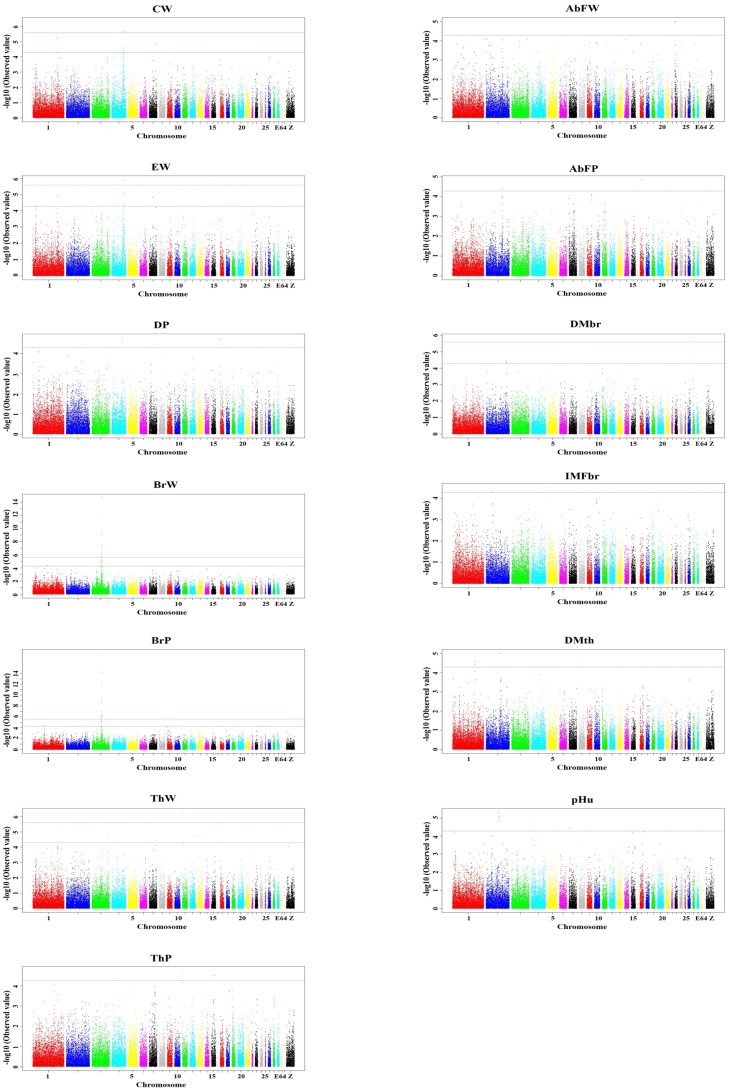
Manhattan plots showing association of all SNPs with carcass and meat quality traits from compressed MLM. SNPs are plotted on the x-axis according to their position on each chromosome against association with these traits on the y-axis (shown as -log10 p-value). The black dashed line indicates genome-wise significance of suggestive association (p-value = 5.19E−05), and the red dashed line shows genome-wise 5% significance with a p-value threshold of 2.59E−06. Abbreviations: CW, carcass weight; EW, eviscerated weight; DP, dressed percentage; BrW, breast muscle weight; BrP, percentage of breast muscle; ThW, thigh muscle weight; ThP, Percentage of thigh muscle; AbFW, weight of abdominal fat; AbFP, percentage of abdominal fat; DM_Br,_ dry matter content in breast;IMF_Br_, intramuscular fat in breast; DM_Th_, dry matter content in thigh; pHu, ultimate pH.

The results for all SNPs demonstrated to have genome-wide significance (P<2.59E−06) or suggestive significance (P<5.19E−05) are presented in full in Table S1 and Figure 1. For the traits examined, emphasis is placed on the associations revealed by the compressed MLM analyses because the population structure effect shown under the GLM model could be controlled effectively when the compressed MLM was used (Figure S1). Of the SNPs revealed by the compressed MLM model, status of SNPs that reached genome-wide significance exposed by the simple model were also indicated.

### Loci and Genes for Traits Related to Body Composition

#### Carcass Weight (CW)

As detailed in Table 5, there were 10 SNPs that were significantly associated with CW from the compressed MLM, of which six were of genome-wide significance by analysis with MLM and/or GLM. Seven of the SNPs on chicken (Gallus gallus) chromosome 4 (GGA4) were clustered within a 1.06 Mb region (between 78,475,066 bp and 79,531,679 bp), and are located either within or 19 kb–236 kb away from the nearest known genes: ligand dependent nuclear receptor corepressor-like protein (*LCORL*), leucineamino peptidase 3 (*LAP3*), quinoid dihydro pteridine reductase (*QDPR*); LIM domain-binding protein 2 (*LDB2*), transmembrane anterior posterior transformation1 (*TAPT1*). The two SNPs on GGA1 are located within dachshund homolog 1 (Drosophila) (*DACH1*).

**Table 5:** SNPs with genome-wide and suggestive significance for carcass weight, eviscerated weight and dressed percentage traits.

Trait^1^	SNP^2^	Chr.	Position (bp)	Nearest Gene^3^	Distance (kb)	*P*_value	*P*_value_Bonferroni
**CW**	GGaluGA051980	1	161061660	*DACH1*	within	4.86E−05	0.937
	***^b^Gga_rs13964189***	1	161126313	***DACH1***	within	6.34E−06	0.122
	***^ab^Gga_rs15619099***	4	78475066	***LCORL***	236	2.28E−06	0.044
	***^a^GGaluGA265949***	4	78476680	***LCORL***	234	2.06E−06	0.040
	Gga_rs14707179	4	78899898	*LAP3*	19	3.98E−05	0.768
	***^ab^Gga_rs16436561***	4	78993342	***QDPR; LDB2***	103;108	4.85E−07	9.36E−03
	***^b^Gga_rs15619411***	4	79191496	***LDB2***	within	2.11E−05	0.407
	Gga_rs14491543	4	79484730	*TAPT1*	65	3.10E−05	0.598
	***^b^Gga_rs14491627***	4	79531679	***TAPT1***	112	4.79E−05	0.924
	***^b^Gga_rs14627660***	7	33957148	***ARHGAP15***	373	1.27E−05	0.246
**EW**	Gga_rs13964189	1	161126313	*DACH1*	within	1.18E−05	0.228
	Gga_rs15619099	4	78475066	*LCORL*	236	9.92E−06	0.191
	GGaluGA265949	4	78476680	*LCORL*	237	9.65E−06	0.186
	***^b^Gga_rs14707179***	4	78899898	***LAP3***	19	3.81E−05	0.735
	***^b^Gga_rs16436561***	4	78993342	***QDPR; LDB2***	103;108	1.21E−06	0.023
	***^b^Gga_rs15619411***	4	79191496	***LDB2***	within	8.74E−06	0.169
	***^b^Gga_rs14491543***	4	79484730	***TAPT1***	65	4.51E−05	0.870
	***^b^Gga_rs14611966***	7	19419046	***DYNC1I2***	6	1.47E−05	0.284
	Gga_rs15043317	19	211349	*CDD*	27	4.16E−05	0.802
**DP**	Gga_rs14487406	4	70644714	*UCHL1*	92	2.92E−05	0.563
	Gga_rs15613971	4	70769617	*CHRNA9*	102	1.56E−05	0.300
	Gga_rs14104932	17	919605	*FBXW5*	8	2.08E−05	0.400

Note: ^1^CW, carcass weight; EW, eviscerated weight; DP, dressed percentage;

2 SNPs with superscript “a” were of genome-wide significance by compressed MLM, those with superscript “b” were significant by GLM, and those with superscript “ab” were significant by both methods; these SNPs are shown underlined in ***boldface***;

3 The nearest known gene to the genome-wide significant SNPs are shown underlined in ***boldface***.

#### Eviscerated Weight (EW)

In the case of EW, nine significant SNPs were identified by compressed MLM, of which five were of genome-wide significance by GLM analysis. The six SNPs on GGA4 are the same as those found for CW but there were slight differences in which of them were of greater significance (Table 5). The one SNP on GGA1, within *DACH1*, was also the same as that associated with CW. The remaining two SNPs were on GGA7 and GGA19, neither was associated with any other trait.

#### Dressed Percentage (DP) and Percentage of Eviscerated Weight (EWP)

As seen from Table 5, three SNPs with suggestive significance for DP were identified by the two methods. The two SNPs on GGA4 were in the vicinity (within 92 and 102 kb) of ubiquitin carboxy-terminal hydrolase L1 (*UCHL1*) and neuronal acetylcholine receptor subunit alpha-9 (*CHRNA9*). The SNP on GGA17 was in close proximity (8 kb) to F-box/WD repeat-containing protein 5 (*FBXW5*). No SNPs associated with the percentage of eviscerated yield (EWP) were found.

#### Breast muscle Weight (BrW) and Percentage (BrP)

Associations identified with these breast muscle traits are shown in Table 6. All 19 SNPs on GGA3 were detected as being significantly associated with BrW (most at the genome-wide level) and clustered within a 5.74 Mb region (61,828,480 bp −68,570,699 bp). There were 15 SNPs, within a similar region, significantly associated with BrP; 11 were common to the two obviously related traits. Most of the SNPs located within or near RNA methyltransferase 11 (*TRMT11*), nuclear receptor coactivator (*NCOA7*), tumor protein D53 (*TPD52L1*), fatty acid binding protein 7 (*FABP7*) and gap junction protein, alpha 1 (*GJA1*) genes. Noteworthy are the SNPs with extreme signals (P = 2.37E−15 and 9.78E−13 for BrW and 1.98E−18 and 6.12E−15 for BrP) located near *FABP7* and *GJA1*, accounting for more than 8% of the phenotypic variance of BrW and BrP (Table S1).

**Table 6:** SNPs with genome-wide and suggestive significance for breast muscle weight and percentage of breast muscle.

Trait^1^	SNP^2^	Chr.	Position	Nearest Gene^3^	Distance (kb)	*P*_value	*P*_value_Bonferroni
**BrW**	Gga_rs14365357	3	61828480	*TRMT11*	307	2.84E−06	0.055
	***^a^GGaluGA224777***	3	62085586	***TRMT11***	50	7.59E−07	0.015
	Gga_rs15367914	3	62189978	*NCOA7*	within	1.06E−05	0.204
	***^a^GGaluGA224820***	3	62206992	***NCOA7***	within	2.50E−06	0.048
	***^a^Gga_rs14735513***	3	62636566	***TPD52L1***	51	4.40E−10	8.48E−06
	GGaluGA224956	3	62693188	*TPD52L1*	108	8.46E−06	0.163
	GGaluGA224987	3	62817611	*TPD52L1*	232	2.16E−05	0.417
	***^a^Gga_rs16286357***	3	63552711	***FABP7***	290	2.37E−15	4.56E−11
	***^a^Gga_rs14366273***	3	63744854	***FABP7***	98	4.75E−08	9.15E−04
	Gga_rs16286470	3	63784515	*FABP7*	58	5.09E−06	0.098
	***^a^GGaluGA225255***	3	64382440	***GJA1***	27	4.93E−08	9.50E−04
	***^a^Gga_rs16287013***	3	64403287	***GJA1***	6	4.93E−08	9.50E−04
	***^a^Gga_rs14366866***	3	64788921	***GJA1***	371	9.78E−13	1.89E−08
	***^a^Gga_rs14366948***	3	64891040	***GJA1***	473	5.73E−07	0.011
	***^a^Gga_rs16287534***	3	64938719	***ASF1A***	498	8.16E−10	1.57E−05
	***^a^GGaluGA225384***	3	65001206	***ASF1A***	436	9.94E−08	1.92E−03
	GGaluGA226040	3	66996816	*HDAC2*	449	3.99E−05	0.768
	***^a^Gga_rs14369404***	3	67826596	***LOC396473***	307	2.41E−06	0.047
	Gga_rs13690373	3	68570699	*FYN*	38	1.19E−05	0.229
**BrP**	***aGGaluGA224327***	3	59817231	*gene desert*	−	1.37E−06	0.025
	***^a^GGaluGA224777***	3	62085586	***TRMT11***	50	7.98E−07	0.015
	***^a^Gga_rs14735513***	3	62636566	***TPD52L1***	51	1.54E−12	2.98E−08
	***^a^GGaluGA224956***	3	62693188	***TPD52L1***	108	1.08E−07	2.08E−03
	GGaluGA224987	3	62817611	*TPD52L1*	232	3.29E−06	0.064
	Gga_rs14365946	3	62858153	*TPD52L1*	273	3.36E−06	0.065
	***^a^Gga_rs16286357***	3	63552711	***FABP7***	290	1.98E−18	3.82E−14
	***^a^Gga_rs14366273***	3	63744854	***FABP7***	98	8.63E−10	1.66E−05
	***^a^Gga_rs16286470***	3	63784515	***FABP7***	58	4.85E−07	9.35E−03
	***^a^GGaluGA225255***	3	64382440	***GJA1***	27	5.81E−10	1.12E−05
	***^a^Gga_rs16287013***	3	64403287	***GJA1***	6	5.81E−10	1.12E−05
	***^a^Gga_rs14366866***	3	64788921	***GJA1***	371	6.12E−15	1.18E−10
	***^a^Gga_rs14366948***	3	64891040	***GJA1***	473	1.24E−06	0.024
	***^a^Gga_rs16287534***	3	64938719	***ASF1A***	498	3.01E−09	5.81E−05
	***^a^GGaluGA225384***	3	65001206	***ASF1A***	436	6.34E−07	0.012

Note: ^1^BrW, breast muscle weight; BrP, percentage of breast muscle weight;

2 SNPs with superscript “a” were of genome-wide significance by compressed MLM, those with superscript “b” were significant by GLM, and those with superscript “ab” were significant by both methods; these SNPs are shown underlined in ***boldface***;

3 The nearest known gene to the genome-wide significant SNPs are shown underlined in ***boldface***.

#### Thigh muscle Weight (ThW) and Percentage (ThP)

Four SNPs, significantly associated with ThW were identified by compressed MLM and occurred on GGA3, GGA11, GGA2 and GGA16; those on GGA3 and GGA11 were of genome-wide significance (Table 7). The SNP on GGA3 was also associated with LW, though of slightly lesser significance. The significant SNP on GGA11was close (8 kb) to nuclear transport factor 2 (*NUTF2*) and this locus was also associated with ThP. The other SNP for ThP was of genome-wide significance and was on GGA2, just 2 kb from forkhead box N4 (*FOXN4*).

**Table 7:** SNPs with genome-wide and suggestive significance for thigh muscle weight, percentage of thigh muscle, weight of abdominal fat and percentage of abdominal fat traits.

Trait^1^	SNP^2^	Chr.	Position	NearestGene^3^	Distance(kb)	*P*_value	*P*_value_Bonferroni
ThW	***^b^Gga_rs14402759***	3	102055329	***MYCN***	59	1.40E−05	0.270
	***^ab^Gga_rs14017512***	11	909864	***NUTF2***	8	5.27E−07	0.010
	GGaluGA086699	12	13524309	*PTPRG*	145	1.92E−05	0.371
	Gga_rs15788030	16	138076	*TRIM39*	within	4.32E−05	0.833
ThP	Gga_rs14017512	11	909864	*NUTF2*	8	1.69E−05	0.327
	***^b^GGaluGA108614***	15	6871802	***FOXN4***	2	3.07E−05	0.591
AbFW	***^b^Gga_rs16186066***	23	519991	***PUM1***	11.7	1.09E−05	0.209
	***^b^GGaluGA187223***	23	525122	***SNRNP40***	14.8	9.90E−06	0.191
AbFP	***^b^Gga_rs14228798***	2	106553422	***ZNF521(H)***	209.8	4.03E−05	0.799
	Gga_rs15030858	17	6149544	*ASB6*	51.6	1.41E−05	0.279

Note: ^1^ThW, thigh muscle weight; ThP, percentage of thigh muscle; AbFW, weight of abdominal fat; AbFP, percentage of abdominal fat;

2 SNPs with superscript “a” were of genome-wide significance by compressed MLM, those with superscript “b” were significant by GLM, and those with superscript “ab” were significant by both methods; these SNPs are shown underlined in ***boldface***;

3 The nearest known gene to the genome-wide significant SNPs are shown underlined in ***boldface***.

#### Weight and Percentage of Abdominal Fat (AbFW, AbFP)

Two SNPs, of genome-wide significance for AbFW by GLM, were found on GGA23; one in the vicinity (14.8 kb away) from small nuclear ribonucleoprotein 40 kDa (U5) (*SNRNP40*) and the other was 11.7 kb from pumilio homolog 1 (*PUM1)*. Of the two SNPs identified by compressed MLM to be associated with AbFP, the one on GGA2 was of genome-wide significance by GLM and was 209.8 kb from a human reference gene, human zinc finger protein 521 (*ZNF521*), with 86.4% sequence identity with the chicken genome. The other detected SNP, on GGA17, had proximity (within 51.6 kb) to ankyrin repeat and SOCS box protein 6 (*ASB6*) (Table 7).

### Loci and Genes for Meat Quality Traits

The SNPs associated with four traits related to meat quality are provided in Table 8; no SNPs were significantly associated with intramuscular fat content of thigh (IMF_Th_) and shear force (SF) of breast muscle.

**Table 8:** SNPs with genome-wide and suggestive significance for meat quality traits.

Trait^1^	SNP^2^	Chr.	Position	NearestGene^3^	Distance(kb)	*P*_value	*P*_value_Bonferroni
**DM_Br_**	Gga_rs16137527	2	136845425	*ANGPT1*	96.9	4.5E−05	0.862
	GGaluGA169253	2	137046977	*ANGPT1*	Within	3.7E−05	0.712
	GGaluGA169274	2	137165373	*ANGPT1*	33.2	3.7E−05	0.712
	Gga_rs15238188	27	1784703	*FTSJ3*	177.5	1.0E−05	0.192
	***^ab^Gga_rs16205470***	27	1822879	***FTSJ3***	215.7	1.4E−06	0.028
**IMF_Br_**	Gga_rs14173354	2	43728839	*CCK*	120.9	4.80E−05	0.926
	Gga_rs16470339	5	15951489	*TOLLIP*	159.7	2.98E−05	0.575
**DM_Th_**	Gga_rs15447898	1	146173970	*gene desert*	−	2.64E−05	0.508
	Gga_rs14897593	1	146262528	*gene desert*	−	2.64E−05	0.508
	Gga_rs13949933	1	146286933	*gene desert*	−	3.69E−05	0.711
	Gga_rs13711523	1	146304286	*gene desert*	−	2.64E−05	0.508
	Gga_rs16070025	2	95730386	*SOCS6*	208.4	9.98E−06	0.192
	GGaluGA329954	8	22682464	*CMPK1*	8.5	1.95E−05	0.376
**pHu**	GGaluGA155419	2	83950900	*gene desert*	−	4.92E−06	0.098
	GGaluGA155426	2	83978295	*gene desert*	−	4.92E−06	0.098
	Gga_rs15121008	2	84587977	*gene desert*	−	1.38E−05	0.274
	Gga_rs15121015	2	84712690	*gene desert*	−	1.38E−05	0.274
	Gga_rs15121029	2	84862485	*gene desert*	−	1.38E−05	0.274
	Gga_rs15121036	2	84947884	*gene desert*	−	7.98E−06	0.158
	Gga_rs15121102	2	85115430	*gene desert*	−	1.38E−05	0.274
	Gga_rs16050844	2	86015253	*GALNT1*	66.6	9.80E−06	0.194
	Gga_rs14422922	4	5263499	*PCDH19*	Within	4.89E−05	0.969
	Gga_rs15483905	4	6275672	*DIAPH1*	286.3	1.04E−05	0.207
	Gga_rs14603569	7	5742651	*SPP2*	143.9	3.54E−05	0.701

Note: ^1^DM_Br_, dry matter content in breast muscle; IMF_Br_, intramuscular fat content in breast; DM_Th_, dry matter content in thigh muscle; pHu, ultimate pH;

2 SNPs with superscript “a” were of genome-wide significance by compressed MLM, those with superscript “b” were significant by GLM, and those with superscript “ab” were significant by both methods; these SNPs are shown underlined in ***boldface***;

3 The nearest known gene to the genome-wide significant SNPs are shown underlined in ***boldface***.

#### Dry Matter content in Breast (DM_Br_)

Five SNPs of significance were identified, located on GGA2 and GGA27. The three SNPs on GGA2 are in the vicinity of or within angiopoietin 1 (*ANGPT1*). The SNPs on GGA27 have some proximity to FtsJ homolog 3 (*E. coli, FTSJ3*); SNP Gga_rs16205470 was the only SNP related to meat quality shown to have genome-wide significance.

#### Intramuscular Fat in Breast (IMF_Br_)

Two significant SNPs were identified and located on GGA2 and GGA5. The SNP on GGA2 is 120.9 kb away from cholecystokinin (*CCK*). The SNP on GGA5 is 159.7 kb from Toll interacting protein (*TOLLIP*).

#### Dry Matter content in Thigh (DM_Th_)

Six significant SNPs associated with DM_Th_ were identified and located on GGA1, GGA2 and GGA8. The SNP on GGA2 is 208.4 kb away from suppressor of cytokine signaling 6 (*SOCS6*). The SNP on GGA8 is 8.5 kb from UMP-CMP kinase (*CMPK1*).The four SNPs on GGA1 are clustered within a 1.30 Mb region with no annotated genes nearby.

#### Ultimate pH (pHu)

Eleven significant SNPs were identified and located on GGA2, GGA4 and GGA7. The eight SNPs on GGA2 are distributed within a 2.06 Mb region and only one gene (Polypeptide N-acetylgalactosaminyltransferase 1, *GALNT1*) is in the vicinity. Two SNPs on GGA4 located within protocadherin 19 (*PCDH19*) or 286.3 kb away from diaphanous homolog 1 (*DIAPH1*). The SNP on GGA7 located 143.9 kb from the secreted phosphoprotein 2 (*SPP2*).

### Validation of Candidate Genes for BrW and BrP from GWAS by Q-PCR

**Figure 2:**
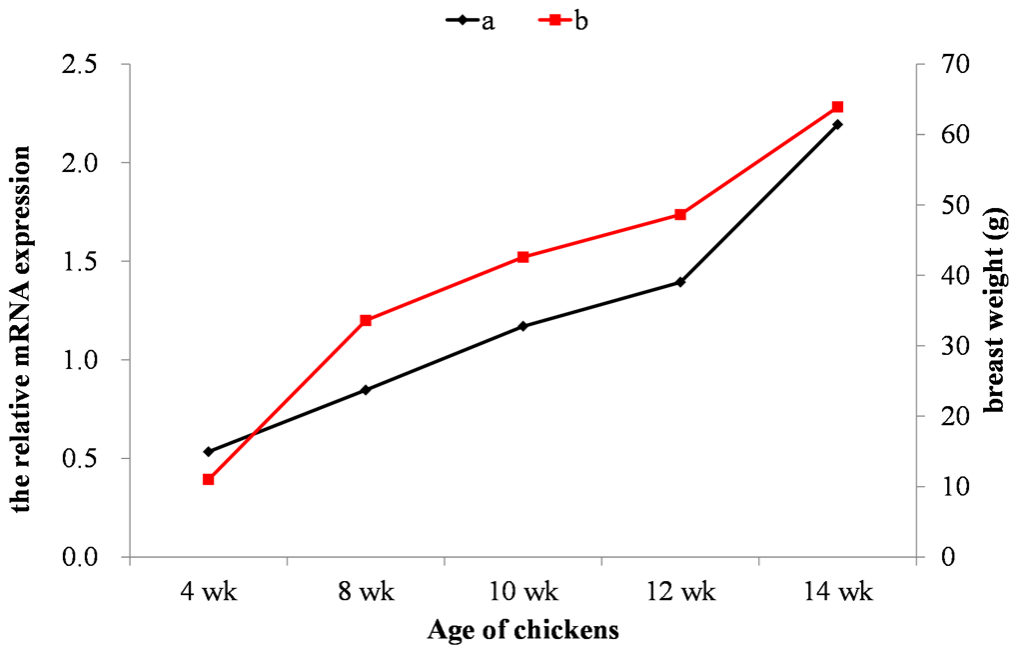
The relative mRNA expression of gap junction protein, alpha 1 gene (a) was consistent with that of the breast muscle weight (b) across development. The mRNA expression is shown as the number of copies (×10^5^) per µg total RNA; Data are means (n = 6).

Expression of candidate genes detected near associated signals for BrW and BrP in the GWAS analysis was further tested by real-time quantitative PCR (Q-PCR) in breast muscle. After measuring the mRNA content of five genes (*NCOA7*, *TPD52L1*, *FABP7*, *GJA1*, *ASF1A*) located in a 0.65 Mb region for BrW and BrP, the change in *GJA1* expression was found to be consistent with that of the breast muscle weight across development (4, 8, 10, 12 and 14 week) (Figure 2). The correlation between *GJA1* mRNA and BrW was moderate (r = 0.554) and significant at the 0.001 level. It is highly possible that *GJA1* is a functional gene for breast muscle development in chickens.

## Discussion

### Genome-wide Association Analysis

To identify potential loci and candidate genes affecting chicken body composition and meat quality traits, GWAS studies were performed using a conservation population of Beijing-You (BJY) chickens. The BJY chicken is one representative indigenous breed in China [16], a color-feathered, slow-growing chicken and has superior quality of meat products [17], [18]. Most of the traits tested showed considerable ranges between maximal and minimal values (Table 3), as would be expected in a population being maintained for the conservation of genetic diversity; this variability would be expected to increase the power of the GWAS.

Two statistical methods, compressed mixed linear model (MLM) and generalized linear model (GLM), were implemented to analyze association between SNPs and phenotypes. Emphasis is placed on the associations revealed by the compressed MLM analyses because population structure effect could be controlled and false positives could be reduced with this approach, as shown in Q-Q plots (Figure S1). However, because the degree of association might be reduced in MLM [19], so of the SNPs revealed by compressed MLM, status of SNPs that reached genome-wide significance exposed by the simple model were also indicated. In addition, because many SNPs reaching suggestive association in both MLM and GLM located within similar regions to those with genome-wide significance, it is proposed that SNPs with suggestive association also indicate important loci.

### Loci and Genes for Traits Related to Body Composition

Breast muscle yield is the most important carcass component in meat-type chickens because of the high premium paid by consumers. Of special interest, one important region (61.83 Mb–68.57 Mb) on GGA3 was identified as being associated with BrW and BrP. Most of the SNPs were located within or near tRNA methyltransferase 11 (*TRMT11*), nuclear receptor coactivator (*NCOA7*), tumor protein D53 (*TPD52L1*), fatty acid binding protein 7 (*FABP7*) and gap junction protein, alpha 1(*GJA1*) genes. Of these five genes, only the change of *GJA1* (gap junction protein, alpha 1) expression was consistent with that of BrW across development (4, 8, 10, 12 and 14 week) and the correlation between mRNA level and BrW was significant (p<0.001). Thus, it is highly possible that *GJA1* is a functional gene for chicken breast muscle development. This result might supply a novel functional gene for breast muscle development and extent the known function of *GJA1* which has been found to play a role in skeletal form [20], [21]. In practical breeding programs, birds could be selected for breeding stock based on the desired allele in *GJA1* for BrW and BrP.

For CW and EW, one consistent region was identified (about 78.47 Mb to 79.53 Mb) on GGA4, which corresponded to QTL regions previously reported [22], [23]. There were seven significant SNPs located near four functional genes (*LCORL*, *LAP3*, *LDB2*, and *TAPT1*). Polymorphisms in *LCORL* have been detected to associate with human skeletal frame size, linear growth [24]–[27] and feed intake and growth of cattle [28]. The LIM domain-binding factor 2 (*LDB*2) has been associated with chicken body weight (7–12 wk) and average daily gain (6–12 wk) in another GWAS study [15]. The transmembrane protein, TAPT1, involved in transporting molecules across membranes, was speculated [29] to be a downstream effector of HOXC8 that may relate to axial skeletal patterning during development. The amino peptidase LAP3 catalyzes the removal of amino acids and peptides as part of protein maturation and degradation [30].

On GGA1, significant SNPs associated with both CW and EW were found within *DACH1* and this gene located near a QTL region known to be related to CW [31]. The gene *DACH1* is a target of FGF signaling during limb skeletal development [32]. In addition, two significant SNPs located in GGA3 were found to relate with LW and were also in the QTL region for 40d body weight and 21d body weight, respectively [33], [34].

The SNP Gga_rs14402759 on GGA3 was found to associate with both ThW and LW, and this locus was near *MYCN.* These two associations are consistent with the relatively high correlation (r^2^ = 0.75) found between two traits, which possibly reflects their being controlled by common loci.

### Loci and Genes for Traits Related to Meat Quality Traits

Three significant SNPs on GGA2 were found to associate with DM_Br_ and were located in the vicinity of or within *ANGPT1.* Angiopoietin-1 (ANGPT1) is an angiogenesis factor that is also an important modulator of skeletal muscle function [35]. Angiopoietin-1 (ANGPT1) is mainly produced by cardiac, skeletal and smooth muscle cells, and adventitial cells [36]. The additional two SNPs on GGA27 were near *FTSJ3* (a putative ortholog of yeast Spb1p) and conditional knockdown revealed that depletion of *FTSJ3* affects HEK293 cell proliferation and causes pre-rRNA processing defects [37].

For DM_Th_, four SNPs of suggestive significance on GGA1 were clustered within a 1.3 Mb region, currently lacking any annotated gene. Another SNP on GGA2 was located near *SOCS6*, which is involved in the regulation of glucose metabolism and plays an important role in regulating insulin action *in vivo* [38].

A SNP on GGA2 was associated with IMF_Br_ and cholecystokinin (*CCK*) was found nearby. The gut hormone CCK plays a multiplicity of roles, including those influencing digestion of fat [39].

For ultimate pH, eight SNPs within a 2.06 Mb region on GGA2 were identified although only one known gene (N-acetylgalactosaminyltransferase 1, *GALNT1*) has been found near this region. The encoded protein catalyzes the transfer of N-acetylgalactosamine (GalNAc) from UDP-GalNAc to the hydroxyl group of a serine or threonine residues on proteins with O-linked glycosylation, a common post-translational modification. Two significant SNPs were found on GGA4, within *PCDH19* and near *DIAPH1*, respectively, and another on GGA7 near *SPP2*. Protocadherin-19 (*PCDH19*) plays a role as an adhesion protein in optic nerve fiber bundling, optic nerve targeting, and/or synapse formation [40]. The gene *DIAPH1* encodes a protein that may have a role in the regulation of actin polymerization in hair cells of the inner ear. The *SPP2* gene encodes a secreted phosphoprotein that is a member of the cystatin superfamily. These proteins play important roles in tumorigenesis, stabilization of matrix metalloproteinases, glomerular filtration rate, immunomodulation, and neurodegenerative diseases.

A number of candidate loci associated with meat quality and fat traits were identified, but common loci or genes for these traits were rare. These results reinforce the notion of complexity in the genetic basis underlying meat quality and fat deposition; to a certain extent, they might be influenced by epigenetic factors [41].

## Supporting Information

Figure S1 **Quantile-quantile (Q-Q) plots of the GLM (red dots) and compressed models (blue dots) for carcass and meat quality traits.** Plotted on the x-axis are the expected p-values under the null hypothesis and on the y-axis are the observed p-values. A: CW, carcass weight; B, EW, eviscerated weight; C, DP, dressed percentage; D, EWP, percentage of eviscerated yield; E, BrW, breast muscle weight; F, BrP, percentage of breast muscle; G, ThW, thigh muscle weight; H, ThP, Percentage of thigh muscle; I, AbFW, weight of abdominal fat; J, AbFP, percentage of abdominal fat; K, DM_Br,_ dry matter content in breast; L, IMF_Br_, intramuscular fat in breast; M, DM_Th_, dry matter content in thigh; N, IMF_Th_, intramuscular fat in thigh; O, pHu, ultimate pH, P, SF, shear force of breast muscle.
(TIF)

Table S1 **Information for all significant SNPs related to body composition and meat quality traits.**
(XLSX)
