# Supplementary figures and images for: Genome-Wide Association Study Identifies Loci and Candidate Genes for Body Composition and Meat Quality Traits in Beijing-You Chickens
Source: PLoS One. 2013 Apr 18;8(4):e61172. doi: 10.1371/journal.pone.0061172 (PMC3630158; doi:10.1371/journal.pone.0061172)

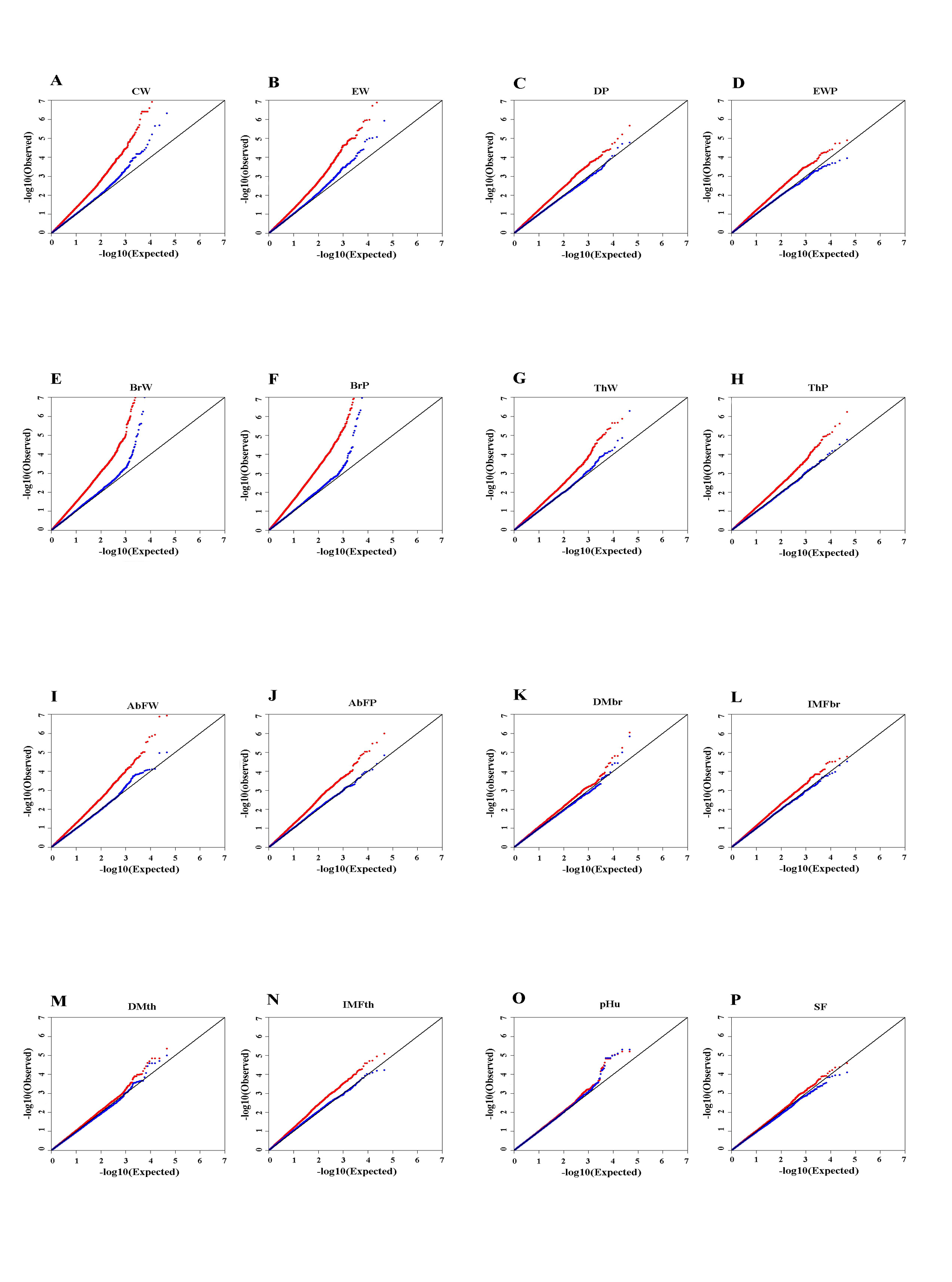

Supplement: Figure S1 — Quantile-quantile (Q-Q) plots of the GLM (red dots) and compressed models (blue dots) for carcass and meat quality traits. Plotted on the x-axis are the expected p-values under the null hypothesis and on the y-axis are the observed p-values. A: CW, carcass weight; B, EW, eviscerated weight; C, DP, dressed percentage; D, EWP, percentage of eviscerated yield; E, BrW, breast muscle weight; F, BrP, percentage of breast muscle; G, ThW, thigh muscle weight; H, ThP, Percentage of thigh muscle; I, AbFW, weight of abdominal fat; J, AbFP, percentage of abdominal fat; K, DMBr, dry matter content in breast; L, IMFBr, intramuscular fat in breast; M, DMTh, dry matter content in thigh; N, IMFTh, intramuscular fat in thigh; O, pHu, ultimate pH, P, SF, shear force of breast muscle. (TIF) [file pone.0061172.s001.tif]
